# Age‐ and sex‐dependent variation in relatedness corresponds to reproductive skew, territory inheritance, and workload in cooperatively breeding cichlids

**DOI:** 10.1111/evo.14348

**Published:** 2021-10-19

**Authors:** Dario Josi, Dik Heg, Tomohiro Takeyama, Danielle Bonfils, Dmitry A. Konovalov, Joachim G. Frommen, Masanori Kohda, Michael Taborsky

**Affiliations:** ^1^ Division of Behavioural Ecology, Institute of Ecology and Evolution University of Bern Hinterkappelen Switzerland; ^2^ Conservation, Ecology, Evolution and Behaviour Research Group, Ecology and Environment Research Centre, Department of Natural Sciences Manchester Metropolitan University Manchester UK; ^3^ CTU Bern University of Bern Bern Switzerland; ^4^ Department of Biosphere‐Geosphere Science Okayama University of Science Okayama Japan; ^5^ Department of Biology and Geosciences, Graduate School of Science Osaka City University Osaka Japan; ^6^ College of Science and Engineering James Cook University Townsville Australia

**Keywords:** Cooperation, alloparental care, multi‐layered society, direct and indirect fitness benefits, *Neolamprologus savoryi*, division of labor, polygyny

## Abstract

Kin selection plays a major role in the evolution of cooperative systems. However, many social species exhibit complex within‐group relatedness structures, where kin selection alone cannot explain the occurrence of cooperative behavior. Understanding such social structures is crucial to elucidate the evolution and maintenance of multi‐layered cooperative societies. In lamprologine cichlids, intragroup relatedness seems to correlate positively with reproductive skew, suggesting that in this clade dominants tend to provide reproductive concessions to unrelated subordinates to secure their participation in brood care. We investigate how patterns of within‐group relatedness covary with direct and indirect fitness benefits of cooperation in a highly social vertebrate, the cooperatively breeding, polygynous lamprologine cichlid *Neolamprologus savoryi*. Behavioral and genetic data from 43 groups containing 578 individuals show that groups are socially and genetically structured into subgroups. About 17% of group members were unrelated immigrants, and average relatedness between breeders and brood care helpers declined with helper age due to group membership dynamics. Hence the relative importance of direct and indirect fitness benefits of cooperation depends on helper age. Our findings highlight how both direct and indirect fitness benefits of cooperation and group membership can select for cooperative behavior in societies comprising complex social and relatedness structures.

Cooperative breeding, where individuals other than breeders provide alloparental care, evolved across many different animal taxa, including insects (Boomsma 2009; Biedermann and Taborsky 2011), crustaceans (Hultgren and Duffy 2012), fishes (Heg and Bachar 2006; Taborsky 2016; Tanaka et al. 2018b), birds (Koenig and Dickinson 2016), and mammals (Solomon and French 1997; Clutton‐Brock 2016). According to Hamilton's rule, selection may favor such alloparental care through both direct or indirect fitness benefits to brood care helpers, depending on their costs and benefits, and the relatedness between actors and beneficiaries (Hamilton 1964; Taborsky 1984; Clutton‐Brock 2002; Stiver et al. 2005; Field and Leadbeater 2016; Komdeur et al. 2017). Kin selection may explain why individuals delay dispersal and help related group members in raising their offspring (Hamilton 1964; Foster et al. 2006; Bourke 2014). Indeed, in several taxa, individuals seem to preferentially cooperate with close relatives (Choe and Crespi 1997; Russell and Hatchwell 2001; Griffin and West 2003; Koenig and Dickinson 2016). However, in many cooperative breeders at least some helpers are not related to the breeding pair producing the offspring to be cared for (Dierkes et al. 2005; Clutton‐Brock 2009; Riehl 2013). These helpers will not gain indirect fitness benefits but are assumed to acquire direct benefits instead, for example, through increased tolerance by dominant individuals allowing them to remain in the group (“pay‐to‐stay”, Gaston 1978; Kokko et al. 2002; Bergmüller and Taborsky 2005; Fischer et al. 2014; Kingma 2017; Naef and Taborsky 2020a, 2020b), which reflects an exchange of different commodities (Quiñones et al. 2016; Taborsky 2016). Continued group membership may benefit helpers, for example, due to reduced mortality risk (Heg et al. 2004, Heg et al. 2005b; Bergmüller et al. 2005; Kingma et al. 2014), shared reproduction (Richardson et al. 2002; Bruintjes et al. 2011; Riehl 2013; Hellmann et al. 2015), or the opportunity to inherit the breeding position in the future (Balshine‐Earn et al. 1998; Stiver et al. 2006; Riehl 2013; Field and Leadbeater 2016; Kingma 2017).

Clarifying relatedness patterns within groups and the interplay of potential direct and indirect fitness benefits of helping in complex animal societies is crucial for a proper understanding of the evolution of apparently altruistic helping behavior. This is a nontrivial challenge, because the received indirect fitness benefits may differ between helpers, depending on the variation in relatedness with the respective receivers of help (e.g., Dunn et al. 1995; Riehl 2013). In contrast, potential direct fitness benefits can arise for related and unrelated individuals alike, which complicates drawing straightforward conclusions about the relative importance of direct and indirect fitness effects of cooperation (Zöttl et al. 2013; Carter et al. 2019).

In fishes, cooperative breeding has been described for approximately 25 lamprologine cichlids endemic to Lake Tanganyika (Taborsky 1994; Heg and Bachar 2006), where it evolved several times independently (Dey et al. 2017; Tanaka et al. 2018b; Ronco et al. 2021). Notably, the cooperatively breeding species in this clade vary greatly in within‐group relatedness levels, ranging from species where most helpers are unrelated to the breeders they support, to others where subordinates usually help their own parents (Awata et al. 2005; Dierkes et al. 2005; Tanaka et al. 2015). For example, large helpers of *N. pulcher* are often unrelated to the breeders they aid (Dierkes et al. 2005; Stiver et al. 2005), whereas helpers of *Neolamprologus obscurus* are typically closely related to the breeders they assist (Tanaka et al. 2015). Hence, in *N. pulcher* indirect fitness benefits of helping are apparently much less important than direct fitness benefits (Jungwirth and Taborsky 2015), and they decline with helper age (reviewed in Taborsky 2016). In contrast, cooperative behavior of subordinates in *N. obscurus* is probably to a larger extent driven by kin selection (Tanaka et al. 2015). This striking divergence of selection mechanisms responsible for apparently altruistic alloparental care among closely related species sharing a common ecology provides unique opportunities to elucidate the significance of relatedness and group structure for the evolution of cooperative behavior in animals. In comparison to mammals and birds, lamprologine cichlids entail the additional benefit of methodological accessibility, as they have rather small home ranges (Heg et al. 2008; Tanaka et al. 2015; Jordan et al. 2016; Josi et al. 2019) and can be observed from a short distance with little disturbance (e.g., Taborsky and Limberger 1981; Maan and Taborsky 2008; Groenewoud et al. 2016; Josi et al. 2020b). Furthermore, most of the cooperatively breeding Lamprologini defend territories throughout the year and therefore can be observed during the reproductive as well as non‐reproductive periods (Brouwer et al. 2005; Josi et al. 2020b). This enables the collection of large sample sizes on social structure, relatedness patterns, and behavior within manageable time.

Here, we studied within and between‐group relatedness, growth, reproductive skew, and workload in the highly social cichlid *Neolamprologus savoryi*. This species breeds in complex groups with breeder males monopolizing one to several breeder females. Each breeder female defends a separate subterritory and may be assisted by subordinate helpers (together referred to as “subgroup”) within the male territory (Heg et al. 2005a; Garvy et al. 2015; Josi et al. 2019, Josi et al. 2020a,b). We sampled breeding groups in two populations and used microsatellite markers to estimate relatedness. Specifically, we asked whether (1) helpers gain indirect fitness benefits by living and helping in kin structured groups and subgroups; (2) helpers gain direct benefits through reproductive share or the chance to inherit the breeding position. For this, we reconstructed the relatedness within the groups and subgroups and (3) asked if breeder and helpers’ investment in territory maintenance, brood care, and territory defense relate to kin structure and the gained direct and indirect benefits. The interplay of direct and indirect benefits on helping behavior and fitness prospects is still not well understood in cooperative breeders. Answering these questions will elucidate the relative importance of direct and indirect fitness benefits in the evolution of alloparental care.

## Materials and Methods

### STUDY POPULATIONS AND SAMPLING

We sampled and genotyped *N. savoryi* groups in two populations at the Zambian coast of Lake Tanganyika: Kasakalawe (“KK”, 8°46.849' S, 31°04.882' E) and Kasenga (“KS”, 8°42.9' S, 31°08.1' E). The KK study site features a rather homogenous sandy plain with rocks (typically ∅10‐40 cm in size) partly submerged in the sand, at 9.0‐11.5 m depth (Heg et al. 2005a; Josi et al. 2019). In contrast, the KS study site is characterized by layers of stones and large boulders (>1 m diameter), interspersed with patches of gravel and shell debris.

All data were collected by SCUBA diving from February to April and October to November 2003. First, the social status and group membership of each individual was determined based on behavioural observations, home ranges, social interactions, and breeding chamber visits (see Heg et al. 2005a). Individuals are highly territorial, and the home ranges of the breeder females’ subgroups do not overlap with each other (Josi et al. 2021). Within subgroups helpers may have private shelters and their home ranges only overlap to a certain extent, but usually not completely. This allowed us to individually identify these fish using a combination of their social status, their home ranges within the marked territories, their body size, and their individual‐specific, unique color patterns, and markings on the head and flanks (cf. Josi et al. 2020b).

Data at KK were collected from 33 breeding groups containing 495 members (33 breeder males, 60 breeder females, 386 helpers (201 males, 152 females, 33 of unknown sex) and 16 offspring (from 10 different groups)), and 21 group‐independents, summing up to a total of 516 individuals. Data at KS were collected from 10 groups containing 61 members (10 breeder males, 15 breeder females, 36 helpers (18 males, 12 females, 6 of unknown sex)) and 1 group‐independent, summing up to 62 individuals in total. Throughout this paper we refer to “groups” as units encompassing all members within the male breeders’ territory (or “harem”), whereas the term “subgroup” refers to the members of a female breeder's territory. Group members were categorized as breeder males, breeder females, helpers (>15 mm SL), and offspring (<15 mm SL; cf. Groenewoud et al. 2016; Tanaka et al. 2016). “Independents” were fish living singly or in small groups without a breeder female, but they were usually associated with a specific subgroup and occasionally visited by the respective breeder male.

### GROWTH AND AGE ESTIMATION

All fish were caught using tent and fence nets, and anaesthetised using clove oil (eugenol, 1 part eugenol dissolved in 4 parts 70% ethanol; Kreiberg 2000). Body size (standard length, SL) of all individuals was determined under water either to the nearest 0.5 mm using a measuring board (KK) or to the nearest 0.1 mm using a calliper (KS). Small tissue samples of all individuals were taken from the dorsal fin and stored in 99% ethanol for subsequent DNA analyses. The fish were sexed by close inspection of the genital papilla. For verification that the sex was correctly identified, a subsample of fish (N = 61) from KS was dissected after completion of data collection. Apart from these fish used for calibration, all other fish were released back to their shelter, where they recovered within a few minutes (cf. Josi et al. 2020b).

At KK, all focal fish were caught and measured after the behavioural observations had been conducted (see below). At KS, all focal fish were caught at least 8 days before the behavioural observations started. Additionally, growth rates were estimated from a subsample of fish in this population (N = 38), which were caught a second time at the end of the second field season, within a period of 31 ± 9 days (mean ± SD; range: 18–51 days). Of these fish (9 breeder males (initial SL: 50.5‐66 mm), 11 breeder females (38‐50 mm), 7 helper males (20‐37 mm), 10 helper females (21‐34 mm), and one offspring (13 mm)) the growth function was estimated. We used the Blumberg growth curve of Skubic et al. (2004) to estimate the age (in days) of each individual in the respective population (cf. Dierkes et al. 2005). Growth rates at KS are comparable to the KK population (Josi et al. 2021).

### BEHAVIORAL OBSERVATIONS

Focal observations were performed in the KK population (one 10 min observation per individual: *n* = 18 breeder males, 21 breeder females, 13 helper males, and 2 helper females) and the KS population (usually three 15 min observations per individual that were averaged and multiplied by 2/3 to allow joint analysis with the KK sample; n = 10 breeder males, 15 breeder females, 18 helper males and 14 helper females). Focal fish were selected haphazardly and covered the range of body sizes of group members (average ± SD: 48.1 ± 9.4, range: 23.7–70.2 mm SL, *n* = 114). At the beginning of each observation, the observer remained motionless in front of the territory for some minutes to acclimatize the fish to their presence. Due to the previous data collection on colony structure, the fish were habituated to the presence of an observer and behaved normally shortly after their appearance. We recorded the number of aggressive behaviors (overt aggression: biting and ramming; restrained aggression: lateral displays and opercula spreads; Josi et al. 2020a) against conspecific and heterospecific intruders, the number of visits to the breeding chamber (Josi et al. 2020b), and the frequency of territory maintenance behaviours (digging sand or small debris out of the shelter, Josi et al. 2020b). Comparable helping behaviours are shown by all cooperatively breeding Lamprologini (Heg and Bachar 2006; Taborsky 2016; Tanaka et al. 2018a), and are repeatable within individuals over time and context (Chervet et al. 2011; Le Vin et al. 2011; Hamilton and Ligocki 2012; Kasper et al. 2017). We summarized the behavioral frequencies of territory defence against conspecific and heterospecific intruders, breeding shelter visits, and territory maintenance behaviours of each group member to a composite measure of workload (cf. Balshine et al. 2001; Bruintjes et al. 2010). Behavioral frequencies were rounded to the nearest integer value to allow fitting Poisson GLMMs (see “Statistical analysis”).

### MICROSATELLITE ANALYSES

Details on DNA extraction, amplification and analyses, as well as references to the 13 microsatellite loci are provided in the Appendix. Ten loci were used for the KK population and 13 loci for the KS population.

### STATISTICAL ANALYSIS

All statistical analysis was conducted with SPSS26 and R (R Core Team 2017). Growth rate (mm/day) was correlated to ln[SL] in a GLM to account for exponentially diminishing growth over size (both effects of status ‐ breeder or helper, *P* = 0.78; and sex ‐ male or female, *P* = 0.78 were non‐significant). The intercept and slope were used to calculate the average relative age of an individual of a specific body size. This method has been shown to provide reliable age estimates in closely related, similar‐sized lamprologine cichlids (Jungwirth et al. in revision; Skubic et al. 2004). Group size and subgroup size were defined as the number of group members larger than 15 mm SL, including breeders and helpers (Heg et al. 2005a; Josi et al. 2019).

Dyadic estimates of pairwise genetic relatedness (Goodnight and Queller 1999) were calculated using the software *Kingroup* v2.1 (https://github.com/dmitryako/kingroup; Konovalov et al. 2004). Locus TmoM11 was especially prone to genotyping errors and therefore removed for the relatedness estimations (see Appendix Table 1). In the KS population, we additionally removed the loci NP101, Pzeb4, and UNH002 as they deviated from Hardy‐Weinberg equilibrium. Several microsatellite loci contained ‘rare’ alleles (that is, 19.9% of the alleles occurred in one group only), so a bias‐correction procedure to calculate background allele frequencies for each group separately is not recommended (Konovalov and Heg 2008). Therefore, we calculated for each population the population allele frequencies corrected for overall relatedness in *Kingroup* version 2.1, and implemented these as an allele frequency block for the analyses (Konovalov and Heg 2008).

**Table 1:** Number of non‐immigrants (philopatric), possible immigrants, and assured immigrants (‘Immigrant’) per status and sex (% assured immigrants in brackets)

Status, sex	Non‐immigrant	Possible immigrant*	Immigrant	
Breeder male	11	21	1	(3.0)
Breeder female	31	8	21	(35.0)
Helper male	161	7	33	(16.4)
Helper female	126	5	21	(13.8)
Helper unknown sex	30	0	3	(9.1)
Independent male	7	3	7	(41.1)
Independent female	2	0	2	(50.0)
Total	368	44	88	(17.6)

^*^Focal individual has unique genotype inside the group, but philopatry may have been obscured due to rapid turn‐over of breeders and siblings.

The majority of fish caught at KK were genotyped (516 individuals, SL between 6.5 and 65.0 mm; for detailed size distribution see Heg et al. 2005a). The sizes of the 62 fish successfully genotyped at KS (SL between 16.8 and 70.2 mm) were comparable to those genotyped at KK. To correct for potential biotic and abiotic differences between populations that might affect genetic structuring, we tested for population effects on relatedness throughout.

To analyze how relatedness between breeders and helpers varies with helper body size, a General Linear Mixed Model (glmm) was performed. The pairwise genetic relatedness between breeder males and their helpers within the group, as well as the relatedness of breeder females to their helpers in their subgroup, were set as response variables. Helper body size (ln[SL]) was set as a continuous effect and breeder sex as a fixed effect (see also Dierkes et al. 2005). The same analysis was performed on a subset of sexed helpers, that is, by adding helper sex as a fixed effect (excluding n = 120 out of 954 comparisons of helper to breeder relatedness due to uncertain sexing of helpers). Furthermore, we analyzed how the helper relatedness changes with their body size/age. A GLMM was fitted with the pairwise genetic relatedness between helpers of the same subgroup as a response variable and the body size of the larger helper (ln[SL]) as well as the difference in helper size (i.e., ln[SL larger helper minus SL smaller helper], which includes same‐sized helpers), as continuous effects. To test the effect of helper sex, the same analysis was performed on the subset of sexed helpers. The sexes of both helper pairs were included as fixed effects. In both GLMMs ‘group’ was entered as a random effect and “population” as a fixed effect.

Next, we compared the pairwise genetic relatedness of individuals of each social status (breeder males, breeder females, helpers, and independents) among each other with respect to whether individuals were group members of the same subgroup, group members of a different subgroup within the same group, or non‐group members (see Fig. 3). Breeder males were assigned to the subgroup they associated with most of the time. If independents associated with a particular subgroup, they were assigned accordingly. We used the bootstrap data selection and loop macro provided by *spss* 26. Bootstrapping was chosen because this approach is less sensitive to the violation of the assumptions regarding the underlying sampling distribution. We calculated four separate anovas on pairwise genetic relatedness of the bootstrap dataset with Dunnett's C post hoc tests (which does not assume equal variances) for each social status (breeder males, breeder females, helpers, and independents). Every possible combination within each social status was tested against each other category (social status × subgroup member/group member from different subgroup/non‐group member; see also Fig. 3). This was repeated 100 times for each social status. The resulting 100 estimates for *P*‐value and the 95% confidence intervals of the Dunnett's C‐test (lower‐bound and upper‐bound) were averaged. The averaged Dunnett's C‐test results were interpreted as significant if the confidence interval did not include zero.

For the KK population, we furthermore reconstructed parent‐offspring relationships and full‐sib and half‐sib relations contained within each group. The Simpson‐assisted descending ratio algorithm in *Kingroup* version 2.1 (Konovalov 2006) was used to split the data into groups. Once the dataset was split, we checked for parent‐offspring, full‐sib and half‐sib relationships within each group. That was done in *Kingroup* using the likelihood‐method of Goodnight and Queller (1999), where the respective relations were compared against the null hypothesis of no relatedness. The utilized likelihood‐method ensured that if an individual was misplaced by the group‐splitting step, it would be automatically filtered out as a non‐relative by the corresponding *P*‐value of no relatedness. This reconstruction was used threefold: (i) to detect matrilineal or patrilineal inheritance of the group, that is, whether the current breeder had inherited the group; this was possible as usually the other‐sex parent or similar‐aged full or half‐siblings were still present in the group. Nevertheless, this must be regarded as a minimum estimate, because potential mortality of siblings and parents may make inheritance undetectable; (ii) to estimate the minimum and maximum numbers of days each breeder had occupied the breeding position in their respective group, where the minimum is the estimated age of the oldest group member produced (set to zero if no offspring was produced), and the maximum is the estimated age of the next older non‐immigrant (see below) group member not produced by the respective focal breeder. These minimum and maximum estimates were averaged per individual breeder (‘tenure’) and entered into a Kaplan‐Meier life‐table analysis to compare the tenure between breeder males (*n* = 33) and breeder females (*n* = 60). The terminal event was set as ‘censored’ when no next older non‐immigrant group member was available (i.e., the tenure must be regarded as a minimum, “open‐ended” estimate, *n* = 8 breeder males, and 14 breeder females); (iii) to categorize all group members as either non‐immigrants (matrilineal/patrilineal inheritance, or full/half‐siblings present inside the group), possible immigrants (unique genotype, but no older and younger kin‐groups present inside the group to compare against, so rapid breeder turnover may have obscured philopatry), or immigrants (unique genotype plus older and younger kin‐groups present).

Parentage was assigned pairwise using *Kingroup* to all adult group members (potential producers) versus any group member smaller than these adults (potential offspring), taking the growth rates of both fish into account (i.e., whether the potential producer was actually adult at the time the potential offspring was produced). Adulthood was estimated based on benchmark data derived from the dissection of KS fish (*n* = 61): (1) males had developed testes from 32.5 mm SL onward, with clearly developed testes from 48.0 mm SL onwards; (2) females had active ovaries from 31.5 mm SL onward, with the ovaries containing large, developed oocytes from 43.9 mm SL onward. Parentage was assigned based on zero or one mismatch. The latter occurred only among nine breeder males (out of 161) and four breeder females (out of 89) with one of their offspring each. The number of offspring produced by male and female helpers within subgroups was set in relation to their relatedness to the breeders (same or opposite sex) with a Poisson GLMM. Only helpers that were sexually mature (i.e., females larger than 38.5 mm and males larger than 43.5 mm) were included in these analyses (see table 2) and *population* was set as a random effect. The effect of within‐subgroup kinship on the workload (*n* = 114, *n_helpers_* = 50, *n_breeders_* = 64) was tested for breeders and helpers separately using Poisson GLMMs with log‐link and “group” as a random effect. The workload was set as a response variable in all models. For the breeders, we included parentage (number of produced subgroup members), subgroup size, and mean relatedness to all helpers not produced per subgroup, as well as sex (male or female) as a predictor. For helpers, subgroup size, sex, and mean between‐helper relatedness of the respective subgroup (which tests for kinship effects) were included as a predictor. Variables were removed from the model if the difference between the Akaike Information Criterion (AIC) values was equal to or larger than 2.

**Table 2:** Parentage in *N. savoryi*: total number of group members produced inside the focal individual's main subgroup or other subgroups within the same territory, in relation to the total number of group members

			Group members produced in:		Number of group members^b^	
Role and sex	*n* Producers	Body size producers mean (mm) ± SD (range)	Subgroup	Group^a^	Subgroup	Group^a^
Breeder male	31	59.1 ± 5.2 (50.0‐67.9)	109 (32.1%)	18 (11.6%)	340	155
Breeder female	37	45.5 ± 3.9 (40.0‐56.8)	66 (25.1%)	17 (8.5%)	263	199
Helper male	12	52.4 ± 4.8 (43.5‐62.2)	24 (6.1%)	9 (3.1%)	392	292
Helper female	5	41.3 ± 3.5 (38.5‐47.2)	6 (7.9%)	0 (0.0%)	76	37
Independent male	1	52.0	1 (5.0%)	0 (0.0%)	20	27
Independent female	0	−	‐	0 (0.0%)	0	3
Total	86	51.1 ± 7.9 (38.5‐67.9)	206	44		

^a^ In *other* subgroup(s) than the main subgroup in which the focal individual spent most of its time.

^b^ Sample sizes of subgroup or group members (from other subgroup(s)) they could have potentially produced based on age difference of at least 210 days; that is, the focal fish and thus potential parent was approximately 31.0 mm SL or larger at the estimated time point of spawning.

## Results

**Figure 1:**
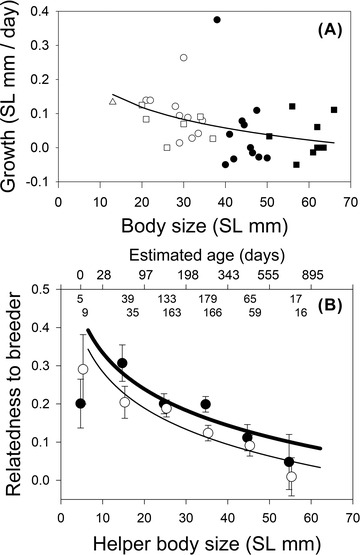
(A) Growth rate per day (mm) of offspring (triangle), helper females (white circles), helper males (white squares), breeder females (black circles), and breeder males (black squares), depending on their initial body size (abscissa). Regression line shows the average body size vs growth rate relationship. Negative values originate from measurement errors within and between observers. (B) Relatedness of the helpers to either the breeder male (black dots, bold line) or breeder female from the same subgroup (white dots, thin line). Depicted are means ± SE per 1 cm size class with the two regression lines from a glmm and estimated age in days (derived from A). Sample sizes are indicated inside the graph.

Growth rate per day significantly declined with body size of the fish (Fig. 1A, β ± SE: intercept 0.38 ± 0.129, *P* = 0.003; slope ‐0.087 ± 0.033, *P* = 0.011). These growth data were used to estimate the relative age of each fish based on its body size. Note that we do not have growth estimates for very large helpers (>40 mm SL), and the two outliers with very high growth rates only marginally influenced the coefficients, which remained significant after the outliers were excluded from the analysis (intercept 0.34, *P* < 0.001; slope −0.081, *P* < 0.001).

### HELPER TO BREEDER RELATEDNESS

Helper to breeder relatedness significantly declined with body size and thus the age of the helpers (Fig. 1B, glmm, data from 41 groups, effect of ln[SL]: *F*_1,945.2_ = 26.5, *P* < 0.0001; estimate ± SE: −0.137 ± 0.027). Helpers were more closely related to the breeder male than to the breeder female (Fig. 1B, glmm: *F*_1,934.8_ = 8.04, *P* = 0.005; estimate ± SE to the males: 0.050 ± 0.018, with relatedness to the females set to zero; intercept ± SE: 0.618 ± 0.108, *F*_1,890.6_ = 42.43, *P* < 0.0001). This effect was similar in both populations (*P* = 0.72). These results did not change when helper sex was included in the model, which by itself showed no significant effect (*P* = 0.34).

### HELPER TO HELPER RELATEDNESS

**Figure 2:**
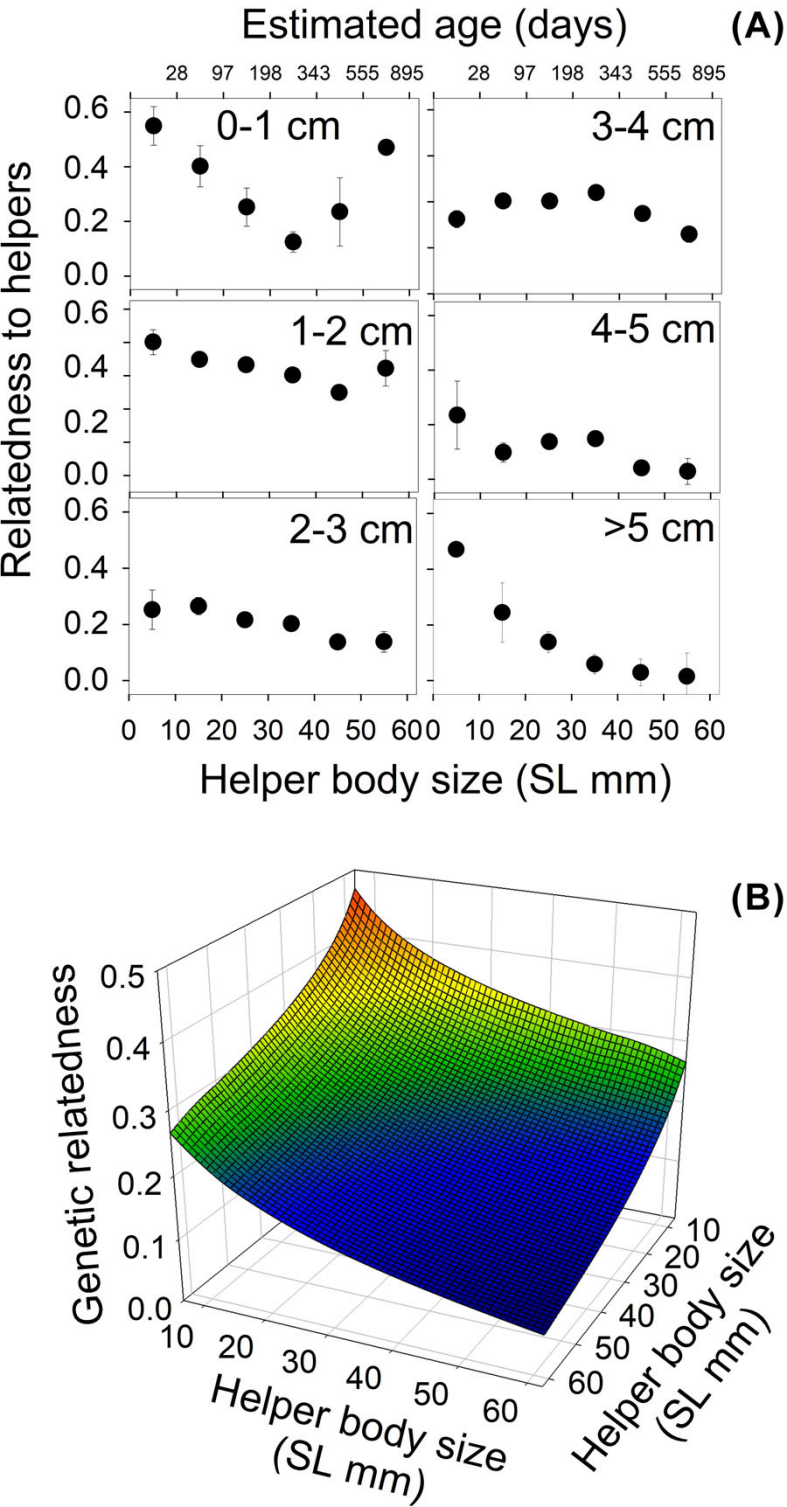
(A) Average relatedness (± SE) of the helpers per 1 cm size class to other subgroup member helpers dependent on body size; also indicated is their estimated age in days. (B) Model predicted estimates were used to depict helper‐to‐helper relatedness within subgroups as a landscape.

Helper to helper relatedness decreased with both absolute helper size (ln[SL]: glmm, (*F*_1,3803.2_ = 80.0, *P* < 0.0001; estimate ± SE: −0.172 ± 0.019) and difference in helper size (ln[difference in SL]: GLMM, (*F*_1,3801.4_ = 42.6, *P* < 0.0001; estimate ± SE: 0.086 ± 0.013), (see Fig. 2A and B). There was no difference between populations (GLMM, *F*_1,36.3_ = 1.5, *P* = 0.22; data of 39 groups; intercept: *F*_1,922.2_ = 123.8, *P* < 0.0001; estimate ± SE: 0.865 ± 0.09). When helper sex was included in the model, it did not reveal significant effects (both *P* = 0.46).

### RELATEDNESS TO GROUP MEMBERS AND SUBGROUP MEMBERS

**Figure 3:**
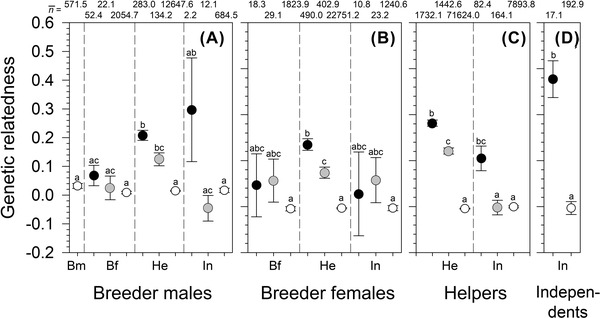
Average relatedness (± SE) of (A) breeder males (Bm), (B) breeder females (Bf), (C) helpers (He), and (D) independents (In) to subgroup members (black dots), group members of a different subgroup (grey dots), or non‐group members (white dots). Note that for breeder males the subgroup was defined as the one where he spent most of his time, and independents were associated with one subgroup only. Note that within each of the four panels, different letters indicate significantly different categories. The sample size of each bootstrap ANOVA per panel with post hoc Dunnett's C‐test was averaged across 100 repetitions and indicated as mean at the top of the graph.

Individuals were more related to members of their own subgroup than to group members belonging to different subgroups, whereas relatedness to non‐group members was virtually zero (see Fig. 3 for average sample sizes per bootstrap anova, 100 bootstrap anovas per status, average *P*‐values: breeder males, *df* = 9, *P* < 0.001; breeder females, *df* = 8, *P* < 0.001; helpers, *df* = 5, *P* < 0.001; independents, *df* = 1, *P* < 0.001). Post hoc bootstrap Dunnett's C‐tests comparing the different categories pairwise were only significant for those combinations where we had reasonable sample sizes (see Fig. 3). These involved mainly combinations with the helpers: (i) Breeder male versus helper relatedness did not differ between group members belonging to the same or different subgroups, and both were significantly higher than relatedness to non‐group members (Fig. 3A). (ii) Breeder female versus helper relatedness declined significantly from group members of the same subgroup to group members from other subgroups, and to non‐group members (Fig. 3B). (iii) Helper vs helper relatedness declined significantly from group members of the same subgroup to group members from other subgroups, and to non‐group members (Fig. 3C). (iv) Helper versus independent relatedness was significantly higher for subgroup members than for both group members from other subgroups and non‐group members (Fig. 3C). Helper versus helper relatedness did not differ from helper vs independent relatedness from the same subgroup (Fig. 3C). (v) Finally, independents were highly related to independents from the same subgroup, whereas relatedness to independents from other groups was zero (Fig. 3D; this result was significant despite the small sample sizes).

### BREEDER TENURE AND TERRITORY INHERITANCE

**Figure 4:**
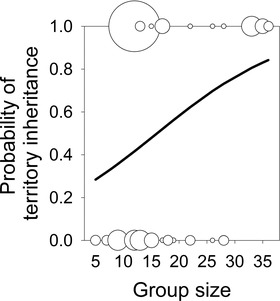
Matrilineal inheritance of groups depending on group size. Multiple cases are indicated by increasing symbol sizes (1 to 10 cases). Also depicted is the model‐predicted logistic regression equation.

The tenure of breeder males (median estimate 257.5 days, *n* = 33; range: 36.5–737 days) was significantly longer than that of breeder females (191.5 days, *n* = 60; range: 11–331 days, Kaplan‐Meier Wilcoxon‐Gehan statistic = 5.3, *P* = 0.021). There was a non‐significant trend that females inherited the breeding territory more often than males (matrilineal inheritance (MLI): 31 out of 60; patrilineal inheritance (PLI): 11 out of 33; χ^2^‐test, χ^2^ = 2.89, *df* = 1, *P* = 0.089). PLI did neither change with group size nor with male body size (Logistic Regression (GLM): intercept: 9.06 ± 6.09; effect of group size, *df* = 1, β ± SE: −0.101 ± 0.094, *P* = 0.28; effect of body size, *df* = 1, β ± SE: −0.156 ± 0.121, *P* = 0.2). In contrast, MLI was more likely to occur in large groups (Fig. 4, Logistic Regression (GLM): intercept: −2.369 ± 5.446; effect of group size, *df* = 1, *P* = 0.04, β ± SE: 0.08 ± 0.039; effect of female body size, *df* = 1, *P* = 0.85, β ± SE: 0.025 ± 0.036).

### IMMIGRATION

**Figure 5:**
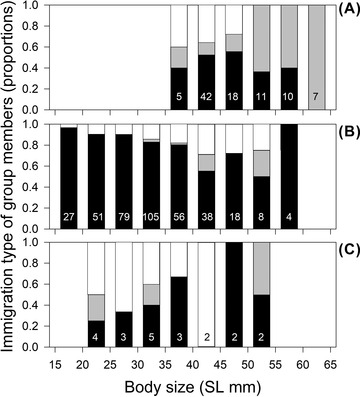
Proportion of non‐immigrants (black), possible immigrants (grey), and immigrants (white) per size class for (A) breeders, (B) helpers, and (C) independents. Sample sizes are indicated inside the bars.

Overall, 16.5% of the group members were immigrants from different groups, with a further 8.6% of individuals being potential immigrants, but their unique genotypes could also have been due to the rapid turnover of breeders and siblings from their own group (Table 1). To investigate the relationship between immigration and status (breeder, helper, or independent) as well as body size, a Multinomial Regression model (MR) was fitted including data of all individuals with known sex (*n* = 467). As a response variable, the immigration state (non‐immigrant, possible immigrant, or immigrant) was included and status as well as body size were set as predictors. The interaction between status and body size was significant (*P* < 0.001), indicating that the relationship between the likelihood of being an immigrant and body size varied depending on the status. Based on this significant interaction we subsequently calculated MRs for each status separately, with the likelihood of being an immigrant as a response variable and body size as a predictor. Larger breeders were less likely to be immigrants (*P* = 0.024, Fig. 5A), whereas larger helpers were more likely to be immigrants than smaller ones (*P* < 0.001, Fig. 5B). There was no relationship between the likelihood of being an immigrant and body size for independents (*P* > 0.1, Fig. 5C). The likelihood of being a “possible immigrant” was higher for both larger breeders and helpers (*P* < 0.001), supporting the interpretation that a failure to resolve the origin of unique genotypes in older individuals may be due to group member turnover, and that some true immigrants may be hidden among these individuals.

### PARENTAGE

We determined either the father (*n* = 121 individuals), the mother (*n* = 49), or both parents (*n* = 40) for individual group members based on one or zero mismatches in the pairwise parent‐offspring assignments. Parentage was skewed toward the dominant pair, but helpers of both sexes had some reproductive share (Table 2). The dominant male and the dominant female produced a significantly higher proportion of the group members inside their “own” subgroup compared to *other* subgroups (Table 2, χ^2^ = 23.3 and 21.0; both df = 1, both *P* < 0.001, *n_males_* = 109_(own)_ vs. 18_(other)_, *n_females_* = 66_(own)_ vs. 17_(other)_). Helper males, but not helper females, also produced offspring in other subgroups (Table 2). The proportion of offspring produced within the own subgroup compared to other subgroups of the same harem did not significantly differ (χ^2^ = 3.4 and 3.1; both df = 1, *P* = 0.066 and 0.079, *n_males_* = 24_(own)_ to 9_(other)_ and *n_females_* = 6_(own)_ to 0_(other)_). Helper males had a higher reproductive share with increasing relatedness to the breeder female (GLMM: n = 48; χ^2^ = 7.51; df = 1, *P* = 0.006). Only few helper females had a reproductive share, which was higher if they were unrelated to the breeder male (GLMM: n = 24; χ^2^ = 5.24; df = 1, *P* = 0.02). The reproductive share of female helpers correlated negatively with their relatedness to the same‐sex dominant (GLMM: n = 24; χ^2^ = 3.8; df = 1, *P* = 0.05), whereas there was no relation between the reproductive share of male helpers and their relatedness to breeder males (GLMM: n = 48; χ^2^ = 0.08; df = 1, *P* = 0.78).

### KINSHIP AND WORKLOAD

**Figure 6:**
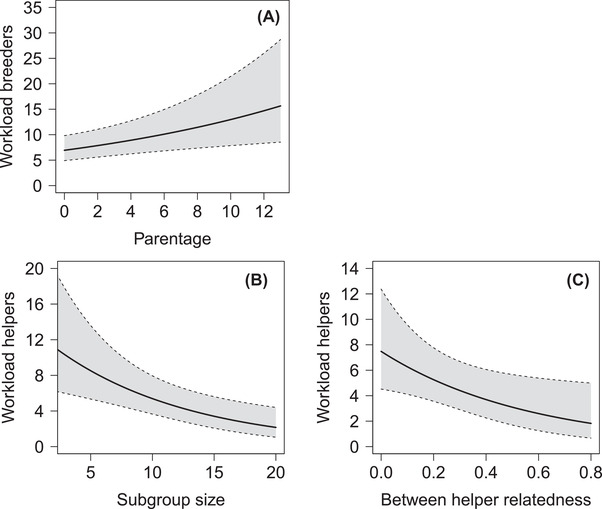
(A) Parentage effects (number of produced subgroup members) on the per capita workload of breeders. Per capita workload of helpers is represented in relation to (B) the size of the subgroups and (C) the relatedness among helpers. The regression lines are based on the final model in Table 3 and predicted 95% confidence intervals are represented in grey.

Overall, the mean kinship within subgroups was significantly higher than zero (*n* = 81 subgroups, *r* = 0.135 ± 0.125 SD, pairwise relatedness values, range: −0.12 ‐ 0.52, one‐sample *t*‐test *t* = 9.72, *df* = 80, *P* < 0.001), but with high variation of relatedness between subgroup members (mean within‐group standard deviation 0.244 ± 0.079 SD, range: 0.01 ‐ 0.57, *n* = 75, excluding six subgroups with only two individuals genotyped). The workload was determined for 64 breeders and 50 helpers. It varied between 1 and 36 behavioral events (rounded to the nearest integer, average ± SD: 8.46 ± 6.05). Breeders worked harder with increasing numbers of own (genetic) offspring present in their subgroup (Fig. 6A, Table 3, β ± SE: 0.063 ± 0.023, *P* = 0.003). Breeder sex (*P* = 0.25), relatedness to helpers not directly produced (*P* = 0.68) and subgroup size (*P* = 0.99) did not affect breeder workload (for details, see Table 3). The helpers’ workload decreased with increasing subgroup size, that is, when there were more individuals sharing duties (Fig. 6B, Table 3, β ± SE: −0.092 ± 0.029, *P* = 0.002). Additionally, with increasing relatedness among helpers of a subgroup, focal helpers worked less (Fig. 6C, Table 3, β ± SE: −1.763 ± 0.795, *P* = 0.02). Overall, male helpers took on a higher workload than female helpers (Table 3).

**Table 3:** Workload and kinship; results of the final model (Poisson GLMM, dependent variable: workload; *n_total_* = 114 focal individuals). Females are the reference for the factor of sex. Significant P‐values are marked in bold

Fixed effects	Estimate	±	SE	*df*	*z*‐value	*P*‐value
Final model breeders:						
Intercept	2.056	±	0.174		11.832	
Parentage	0.063	±	0.022	1	2.888	**0.003**
Sex	0.112	±	0.097	1	1.162	0.25
Relatedness to subgroup helpers	−0.134	±	0.329	1	−0.407	0.68
Dropped variable from full model:						
Subgroup size	0.00006	±	0.018	1	0.003	0.99
Final model helpers:						
Intercept	1.66	±	0.199		8.343	
Subgroup size	−0.092	±	0.029	1	−3.131	**0.002**
Sex	0.43	±	0.152	1	2.837	**0.004**
Relatedness to subgroup helpers	−1.763	±	0.795	1	−2.218	**0.022**

## Discussion

The social and genetic group structure of a population can provide important information about evolutionary mechanisms underlying complex social organization (Cockburn 1998; Riehl 2013; Tanaka et al. 2018b). Here, we show that in a cooperatively breeding cichlid, breeder tenure, territory inheritance, immigration, and reproductive skew are linked to the level of within‐group relatedness, which in turn bears upon the amount of alloparental care provided by subordinate helpers. Group composition in social cichlids is a dynamic process causing relatedness patterns within groups to change with time; the older helpers get, the lower on average is their relatedness to other group members (Dierkes et al. 2005). Due to this dynamic, helper age determines the relative importance of direct and indirect fitness benefits of group living and cooperation.

*N. savoryi* lives in polygynous groups or “harems” divided into subgroups containing female breeders and subordinate helpers. Relatedness differs between group members, declining with the increasing age of helpers (Fig. 1B). Overall, breeder females and helpers were more related to each other in their own subgroup compared to members of other subgroups within the same harem, let alone to members of different groups (Fig. 3). Genetic relatedness of helpers to their breeder male did not differ between the subgroups, but it was higher to the male breeder of their own group than to breeder males of other groups (Fig. 3). Remarkably, helpers were significantly more closely related to their breeder male than to the breeder female of their subgroup (Fig. 1B). This is consistent with the finding that breeder males had a significantly lower turn‐over rate than breeder females (due to death, emigration, or usurpation by unrelated individuals). Helper to helper relatedness declined with their age, and it was significantly higher within than between subgroups (Figs. 2 and 3C).

Dispersal patterns of individuals living in complex social systems are often obscure and difficult to unveil. Recent field evidence suggested that *N. savoryi* helpers may disperse to a neighboring female subgroup within the same male harem (Josi et al. 2021), and a breeder female may establish a subgroup within the group of a closely related male (either the father or the brother; Josi et al. 2019). In line with these findings, the present results indicate that only 16.5% of the group members were immigrants from outside the male's group (or 25.1% if we count all individuals with unique genotypes; see Fig. 5 and Table 1). Immigration was more difficult to detect for larger, that is, older group members (particularly breeders), because the likelihood of finding unique genotypes inside a group increases progressively over time when all close, non‐offspring relatives have disappeared from the group. The low immigration rate implies that both males and females have a good chance to inherit a breeder position within a group. Nevertheless, if we look at the acquisition of breeder status within groups, about 48% of breeder females’ and 67% of breeder males’ positions were acquired by non‐group members, which might indicate that neighboring individuals of both sexes compete with each other, for example, to take over territories of higher quality.

Similar to other cooperative breeders (e.g., Dierkes et al. 2005; Leadbeater et al. 2011) female inheritance was more likely to occur in large groups (see Fig. 4). Such a biased probability of territory inheritance may result from a greater number of potential candidates or a higher survival rate of group members in large groups. It might further indicate that large groups are particularly valuable for breeders as they render higher reproductive rates, enhanced survival for group members, and longer group persistence (Heg et al. 2005b; Jungwirth and Taborsky 2015).

We should like to point out that estimating immigration rates, classification of immigrants, tenure times, and territory inheritance from relatedness patterns may be compromised by reproductive parasitism from outside the group and emigration or death from previous producers of offspring. However, as the relatedness of helpers to the breeder male was higher than to the breeder female within their subgroup, it seems likely that extra‐pair parentage as a potential source of error can be regarded as rather low.

In *N. savoryi*, group membership is essential, as individuals of all sizes face a high risk of predation (Heg et al. 2008; Josi et al. 2020a). Consequently, helpers gain direct fitness benefits through increased protection from predators due to safety in numbers, vigilance of other group members and shared defence, as suggested also in other cooperative breeders (Taborsky 1984; Heg et al. 2004; Caro 2005; Santema and Clutton‐Brock 2013; Groenewoud et al. 2016; Teunissen et al. 2020). Additionally, especially older helpers, can gain further immediate direct fitness benefits by sharing in reproduction (see Table 2), and future benefits by queuing for the breeding position (Griffin and West 2002; Leadbeater et al. 2011; Taborsky 2016; Kingma 2017; for matrilineal inheritance see Fig. 4). Indeed, large helper males sired group members in 20.5% of the cases where the father was successfully detected (*n* = 161), while helper females had produced young in 6.7% of the cases where the mother of the offspring could be successfully detected (*n* = 89). Such reproductive share may also explain why *N. savoryi* helpers assist their breeders in offspring care (Josi et al. 2020a,b). Alternatively, helping can serve as a rent paid to dominants to be allowed to remain in the group (“pay‐to‐stay”; Kokko et al. 2002; Zöttl et al. 2013; Fischer et al. 2014; Naef and Taborsky 2020a, 2020b; Trapote et al. 2021). These results add to the increasing evidence that direct fitness benefits can foster the evolution of cooperative breeding and complex sociality (Clutton‐Brock 2002, 2009; Griffin and West 2002; Riehl 2013; Field and Leadbeater 2016; Komdeur et al. 2016; Taborsky et al. 2016; Kingma 2017; Downing et al. 2018).

The mixed kin‐structure of *N. savoryi* groups and subgroups resembles that of many cooperatively breeding birds and mammals in which complex relatedness and parentage structures have been observed (e.g., Painter et al. 2000; Riehl 2013; Shen et al. 2016; Vitikainen et al. 2017; Leedale et al. 2018; Lukas and Clutton‐Brock 2018). Within cooperatively breeding lamprologine cichlids, the kin structure of *N. savoryi* groups lies between species characterized by high relatedness among group members (e.g., *N. obscurus*; Tanaka et al. 2015) and those featuring groups of largely unrelated individuals (e.g., *Julidochromis ornatus*; Awata et al. 2005). This raises the question which direct and indirect benefits individuals gain from group membership and helping. At one end of the spectrum, helpers mainly gain direct fitness benefits by paying for valuable group membership, participation in reproduction and territory inheritance (*J. ornatus*). At the other end, they may mainly benefit from indirect fitness gains by helping to raise close kin (*N. obscurus*). Hitherto, existing data suffice to estimate the relative importance of direct and indirect fitness effects of delayed dispersal and alloparental care in only few cooperatively breeding vertebrates (Kingma et al. 2011; Jungwirth and Taborsky 2015; Green and Hatchwell 2018). Therefore, *N. savoryi* is a good model to study potential selection mechanisms underlying complex social organization and cooperation. Age seems to be the crucial parameter determining the relative importance of direct and indirect fitness benefits to helpers in *N. savoryi*. Relatedness between helpers and breeders declines with increasing helper age due to breeder mortality and territory takeover of immigrants. Therefore, kin selected benefits are skewed towards young helpers, if they assist their parents in raising their full or half siblings (see Fig. 1B; for a similar situation in *N. pulcher*, see Bruintjes and Taborsky 2011). Young helpers face a particularly high predation risk due to their small size (Heg et al. 2004). Especially these small individuals benefit from remaining with close kin, as breeders invested more in territory defense and brood care when the young in their territory were closely related (see Fig. 6A). Such increased investment in groups containing highly related subordinates further results in additional benefits for helpers, as they are able to reduce their individual workload (Fig. 6C). Finally, relatedness to the dominants might further affect the parentage of the helpers, either through negative effects of inbreeding or through mutual kin selected benefits of co‐breeding. The risk of inbreeding and consequential inbreeding avoidance when the *opposite* sex dominant is a relative may select for increased helper dispersal (Beck et al. 2008; Nelson‐Flower et al. 2012). In contrast, an increase in reproductive opportunities for helpers can select for delayed dispersal, cooperative brood care, and participation in reproduction (Hellmann et al. 2016). Indeed, we found that the reproductive share of helper females declined with increasing relatedness to the breeder male. As helper females always reproduced with males (breeders and subordinates) of the same group, this effect might be explained by inbreeding avoidance. In contrast, male helpers that were related to the dominant female had a higher reproductive share than unrelated males. It is important to note that male helpers did not reproduce with the breeder female in their own group. This suggests that breeder females do not prevent related male helpers from reproducing, which yields indirect fitness benefits to them. From our data, it was not possible to reconstruct the genetic mother of the male helpers’ offspring, which may indicate that the breeder females evicted those mating partners.

A functional relationship between intragroup relatedness and reproductive skew among sexually mature group members has been predicted by transactional skew theory (Reeve and Keller 1997; Johnstone 2000). In particular, concession models predict a positive association between relatedness and reproductive skew. This is based on the logic that if helpers can gain only little indirect fitness benefits by alloparental investment due to low relatedness between donors and beneficiaries, incentives to stay and participate in brood care need to be provided to raise their direct fitness, such as taking a share in reproduction (Vehrencamp 1983; Keller and Reeve 1994). This relationship between relatedness and reproductive skew is compatible with patterns observed in cooperatively breeding lamprologini. For example, in *J. ornatus* and *J. transcriptus* relatedness within groups is low, and male and female helpers gain substantial parentage (41‐56% in *J. ornatus*: Awata et al. 2005; Heg and Bachar 2006; 20–100% in *J. transcriptus*: Kohda et al. 2009). In contrast, in *N. pulcher*, where relatedness within groups is mixed, the share in reproduction of subordinates is much lower (2.5‐19% Dierkes et al. 1999; Heg et al. 2006; Heg and Hamilton 2008; Hellmann et al. 2015). Finally, in *N. obscurus*, where relatedness levels are particularly high within groups, subordinate group members have underdeveloped gonads suggesting reproductive austerity (Tanaka et al. 2015). Similar to *N. pulcher*, *N. savoryi* is apparently positioned in between the extreme cases, with 6–8% of young produced by helpers within their own subgroup. Nevertheless, this rough concordance between the predictions of concession models and the described patterns does not necessarily imply that the underlying hypothesis explains reproductive participation in all lamprologines alike. This note of caution can be illustrated by the existence of outliers from the general trend; in *N. multifasciatus*, for example, preliminary data suggest that high intragroup relatedness goes along with a rather high reproductive share of helpers (Kohler 1998). Hence, it seems that conventional skew models cannot fully account for the complexity of evolutionary mechanisms involved in reproductive skew among members of fish groups (Taborsky 2009).

There is surprisingly little evidence for a positive relationship between intragroup relatedness and reproductive skew in other taxa. In hymenoptera, there are some examples showing the predicted positive relationship between intragroup relatedness and reproductive skew (Reeve et al. 2000; Reeve and Keller 2001; Lucas et al. 2011; Andrade et al. 2016), and the same holds for some birds (Jamieson 1997; Koenig et al. 2009). However, a comparative analysis of all 83 species of cooperatively breeding birds from which genetic data existed suggests that inbreeding avoidance rather than relatedness‐driven reproductive concessions can explain the relationship between kinship and helper reproduction (Riehl 2017). In cooperatively breeding mammals, reproductive skew may be comparatively low because of viviparity hampering full control of reproduction by dominants (Raihani and Clutton‐Brock 2010), and the relationship between intragroup relatedness and reproductive skew may again be driven mainly by inbreeding avoidance (Cooney and Bennett 2000; Nichols 2017; Wikberg et al. 2017). In lamprologine cichlids, dominant group members are largely in control of reproduction due to inherent size differences, and relatedness patterns vary between closely related species sharing the same ecology. Hence, they seem to be a highly suited target for futures studies on the significance of reproductive concessions in the evolution of cooperative breeding and complex social organization. In addition, *N. savoryi* represents a suitable model for studying the ability of brood care helpers to fine‐tune their support to the demands of breeders and to their own costs, as well as to the benefits breeders gain from the presence of helpers in dependence of the relatedness between them.

## AUTHOR CONTRIBUTIONS

D.H., T.T., M.K., and M.T. conceived the study. D.J., D.H., D.B., and D.A.K. analyzed the data; D.H., M.T., D.J., and J.G.F. wrote the manuscript, which was revised and approved by all authors.

## DATA ARCHIVING

Data are archived in the Dryad Digital Repository: https://doi.org/10.5061/dryad.t1g1jwt30.

## ETHICAL NOTE

Data collection followed the ASAB/ABS guidelines for the treatment of animals in behavioural research and teaching, and the regulations of the “Zambian prevention of cruelty to animals” act.

## CONFLICT OF INTEREST

The authors declare no conflict of interest.

Associate Editor: M. E. Maan

Handling Editor: A. G. McAdam

## Microsatellite analyses

Genomic DNA was extracted from ethanol‐preserved fin‐clip samples of approximately 1‐2 mm^2^ in size each using the wizard® Genomic DNA Purification Kit (Catalys Promega AG, Switzerland). DNA was dissolved in 50 μl of DNA Rehydration Solution (provided by the supplier) and stored at ‐20°C until further analysis. DNA was amplified using the qiagen® Multiplex PCR Kit (qiagen AG, Switzerland), thus allowing co‐amplification of several locus‐specific, fluorescently labelled primer pairs in one single PCR reaction (Josi et al. 2019). Fluorescent dyes used as labels at the 5'‐end of forward primers were either 6‐FAM (blue), HEX (green) or NED (yellow). For PCR amplification up to seven microsatellite primer‐pairs were multiplexed in one PCR reaction. PCR reactions were carried out in a 10 μl volume containing 1 μl of genomic DNA, 1x qiagen® Multiplex PCR Master Mix (consisting of qiagen® Multiplex PCR Buffer, 3mM MgCl_2_, dNTP mix and HotStarTaq® DNA Polymerase) and locus‐specific primer pairs with varying concentrations from 0.2 to 0.5 μM according to the intensity of the respective amplification products. Amplification was performed in a geneamp® 9700 PCR System (PE Applied Biosystems) using the following cycling parameters: 15 min. at 95°C, 33 cycles at 94°C for 30 s, 57°C for 90 s and 72° C for 60 s followed by a final step of 72°C for 10 min. Fluorescent PCR fragments were visualized by capillary electrophoresis on an ABI3100® Genetic Analyser (PE Applied Biosystems), automatically analyzed by the genescan® Analysis software version 2.1 (PE Applied Biosystems) and manually revised.

**Table A.1:** Number of adults successfully typed (*n*), number of different alleles (Alleles), observed (*H*_O_) and expected homozygosities (*H*_E_), tests for Hardy‐Weinberg equilibrium (HWE), estimated null allele frequencies (Null), respectively, per locus for the two study populations. Based on the Arlequin 3.01 analysis of adult breeders

Locus	*n*	Alleles	*H*_O_	*H*_E_	HWE	Null	Reference*
*Kasakalawe population (KK)*							
NP007	64	8	0.484	0.52	0.44	0.041	1
NP101	64	16	0.625	0.645	0.36	0.018	2
NP773	64	12	0.734	0.686	0.7	–0.049	1
Pzeb3	64	12	0.516	0.571	0.03	0.041	3
Pzeb4	64	6	0.453	0.514	0.51	0.051	3
TmoM11	64	19	0.563	0.927	<0.001	0.237	4
TmoM25	64	19	0.953	0.911	0.44	–0.027	4
ULI2	64	16	0.563	0.522	0.8	–0.060	1
UME003	64	24	0.875	0.935	0.38	0.028	5
UNH154	64	4	0.109	0.121	1	–0.019	6

*Kasenga population (KS)*							
NP007	19	5	0.526	0.496	1	–0.071	1
NP101	20	10	0.5	0.797	<0.001	0.217	2
NP773	20	5	0.7	0.626	0.8	–0.077	1
Pzeb3	20	4	0.55	0.5	0.84	–0.087	3
Pzeb4	20	4	0.2	0.31	0.01	0.248	3
TmoM11	20	13	0.65	0.917	<0.001	0.154	4
TmoM25	20	16	0.85	0.91	0.17	0.026	4
TmoM27	20	2	0.4	0.37	0.55	–0.110	4
ULI2	20	10	0.65	0.664	0.5	–0.063	1
UME003	20	15	1	0.922	0.43	–0.056	5
UNH002	20	2	0.2	0.31	0.01	0.121	1
UNH106	20	2	0.05	0.099	1	–0.005	7
UNH154	20	2	0.05	0.099	1	–0.005	6

* 1 = Schliewen et al. 2001; 2 = Brandtmann et al. 1999; 3 = Van Oppen et al. 1997; 4 = Zardoya et al. 1996; 5 = Parker et al. 1996; GenBank accession numbers for 6 = G12306 and 7 = G12259.

## References

[evo14348-bib-0001] Andrade, A. C. R. , E. A. Miranda , M. A. Del Lama , and F. S. Nascimento . 2016. Reproductive concessions between related and unrelated members promote eusociality in bees. Sci. Rep 6:26635. 27211350 10.1038/srep26635 PMC4876382

[evo14348-bib-0002] Awata, S. , H. Munehara , and M. Kohda . 2005. Social system and reproduction of helpers in a cooperatively breeding cichlid fish (*Julidochromis ornatus*) in Lake Tanganyika: field observations and parentage analyses. Behav. Ecol. Sociobiol 58:506–516.

[evo14348-bib-0003] Balshine‐Earn, S. , F. C. Neat , H. Reid , and M. Taborsky . 1998. Paying to stay or paying to breed? Field evidence for direct benefits of helping behavior in a cooperatively breeding fish. Behav. Ecol 9:432–438.

[evo14348-bib-0004] Balshine, S. , B. Leach , F. Neat , H. Reid , M. Taborsky , and N. Werner . 2001. Correlates of group size in a cooperatively breeding cichlid fish (*Neolamprologus pulcher*). Behav. Ecol. Sociobiol 50:134–140.

[evo14348-bib-0005] Beck, N. R. , R. Peakall , and R. Heinsohn . 2008. Social constraint and an absence of sex‐biased dispersal drive fine‐scale genetic structure in white‐winged choughs. Mol. Ecol 17:4346–4358. 19378407 10.1111/j.1365-294x.2008.03906.x

[evo14348-bib-0006] Bergmüller, R. , D. Heg , and M. Taborsky . 2005. Helpers in a cooperatively breeding cichlid stay and pay or disperse and breed, depending on ecological constraints. Proc. R. Soc. B 272:325–331. 10.1098/rspb.2004.2960 PMC1634971 15705559

[evo14348-bib-0007] Bergmüller, R. , and M. Taborsky . 2005. Experimental manipulation of helping in a cooperative breeder: helpers ‘pay to stay’ by pre‐emptive appeasement. Anim. Behav 69:19–28.

[evo14348-bib-0008] Biedermann, P. H. W. , and M. Taborsky . 2011. Larval helpers and age polyethism in ambrosia beetles. Proc. Natl. Acad. Sci. U.S.A 108:17064–17069. 21969580 10.1073/pnas.1107758108 PMC3193236

[evo14348-bib-0009] Boomsma, J. J. 2009. Lifetime monogamy and the evolution of eusociality. Philos. Trans. R. Soc. B 364:3191–3207. 10.1098/rstb.2009.0101 PMC2781870 19805427

[evo14348-bib-0010] Bourke, A. F. G. 2014. Hamilton's rule and the causes of social evolution. Philos. Trans. R. Soc. B 369:20130362. 10.1098/rstb.2013.0362 PMC3982664 24686934

[evo14348-bib-0400] Brandtmann, G. , M. Scandura , and F. Trillmich . 1999. Female‐female conflict in the harem of a snail cichlid (*Lamprologus ocellatus*): behavioural interactions and fitness consequences. Behaviour 136:1123–1144.

[evo14348-bib-0011] Brouwer, L. , D. Heg , and M. Taborsky . 2005. Experimental evidence for helper effects in a cooperatively breeding cichlid. Behav. Ecol 16:667–673.

[evo14348-bib-0012] Bruintjes, R. , D. Bonfils , D. Heg , and M. Taborsky . 2011. Paternity of subordinates raises cooperative effort in cichlids. PLoS One 6:e25673. 22022428 10.1371/journal.pone.0025673 PMC3192049

[evo14348-bib-0013] Bruintjes, R. , R. Hekman , and M. Taborsky . 2010. Experimental global food reduction raises resource acquisition costs of brood care helpers and reduces their helping effort. Funct. Ecol 24:1054–1063.

[evo14348-bib-0014] Bruintjes, R. , and M. Taborsky . 2011. Size‐dependent task specialization in a cooperative cichlid in response to experimental variation of demand. Anim. Behav 81:387–394.

[evo14348-bib-0015] Caro, T. M. 2005. Antipredator defenses in birds and mammals. University of Chicago Press, Chicago.

[evo14348-bib-0016] Carter, G. G. , G. Schino , and D. Farine . 2019. Challenges in assessing the roles of nepotism and reciprocity in cooperation networks. Anim. Behav 250:255–271.

[evo14348-bib-0017] Chervet, N. , M. Zöttl , R. Schürch , M. Taborsky , and D. Heg . 2011. Repeatability and heritability of behavioural types in a social cichlid. Int. J. Evol. Biol 321729. 21716729 10.4061/2011/321729 PMC3119426

[evo14348-bib-0018] Choe, J. , and B. J. Crespi . 1997. The evolution of social behaviour in insects and arachnids. Cambridge University Press, Cambridge.

[evo14348-bib-0019] Clutton‐Brock, T. 2002. Breeding together: kin selection and mutualism in cooperative vertebrates. Science 296:69–72. 11935014 10.1126/science.296.5565.69

[evo14348-bib-0020] Clutton‐Brock, T. 2009. Cooperation between non‐kin in animal societies. Nature 462:51–57. 19890322 10.1038/nature08366

[evo14348-bib-0021] Clutton‐Brock, T. 2016. Mammal societies. Wiley Blackwell, Chichester.

[evo14348-bib-0022] Cockburn, A. 1998. Evolution fo helping behavior in cooperatively breeding birds. Annu. Rev. Ecol. Syst 29:141–177.

[evo14348-bib-0023] Cooney, R. , and N. C. Bennett . 2000. Inbreeding avoidance and reproductive skew in a cooperative mammal. Proc. R. Soc. B 267:801–806. 10.1098/rspb.2000.1074 PMC1690596 10819150

[evo14348-bib-0024] Dey, C. J. , C. M. O'Connor , H. Wilkinson , S. Shultz , S. Balshine , and J. L. Fitzpatrick . 2017. Direct benefits and evolutionary transitions to complex societies. Nat. Ecol. Evol 1:0137. 10.1038/s41559-017-0137 28812693

[evo14348-bib-0025] Dierkes, P. , D. Heg , M. Taborsky , E. Skubic , and R. Achmann . 2005. Genetic relatedness in groups is sex‐specific and declines with age of helpers in a cooperatively breeding cichlid. Ecol. Lett 8:968–975. 34517681 10.1111/j.1461-0248.2005.00801.x

[evo14348-bib-0026] Dierkes, P. , M. Taborsky , and U. Kohler . 1999. Reproductive parasitism of broodcare helpers in a cooperatively breeding fish. Behav. Ecol 10:510–515.

[evo14348-bib-0027] Downing, P. A. , A. S. Griffin , and C. K. Cornwallis . 2018. Sex differences in helping effort reveal the effect of future reproduction on cooperative behaviour in birds. Proc. R. Soc. B 285:20181164. 10.1098/rspb.2018.1164 PMC6125912 30135160

[evo14348-bib-0028] Dunn, P. O. , A. Cockburn , and R. A. Mulder . 1995. Fairy‐wren helpers often care for young to which they are unrelated. Proc. R. Soc. B 259:339–343.

[evo14348-bib-0029] Field, J. , and E. Leadbeater . 2016. Cooperation between non‐relatives in a primitively eusocial paper wasp, *Polistes dominula* . Philos. Trans. R. Soc. B 371:20150093. 10.1098/rstb.2015.0093 PMC4760194 26729932

[evo14348-bib-0030] Fischer, S. , M. Zöttl , F. Groenewoud , and B. Taborsky . 2014. Group‐size‐dependent punishment of idle subordinates in a cooperative breeder where helpers pay to stay. Proc. R. Soc. B 281:20140184. 10.1098/rspb.2014.0184 PMC4100499 24990673

[evo14348-bib-0032] Foster, K. R. , T. Wenseleers , and F. L. W. Ratnieks . 2006. Kin selection is the key to altruism. Trends Ecol. Evol 21:57–60. 16701471 10.1016/j.tree.2005.11.020

[evo14348-bib-0033] Garvy, K. A. , J. K. Hellmann , I. Y. Ligocki , A. R. Reddon , S. E. Marsh‐Rollo , I. M. Hamilton , S. Balshine , and C. M. O'Connor . 2015. Sex and social status affect territorial defence in a cooperatively breeding cichlid fish, *Neolamprologus savoryi* . Hydrobiologia 748:75–85.

[evo14348-bib-0034] Gaston, A. J. 1978. The evolution of group territorial behavior and cooperative breeding. Am. Nat 112:1091–1100.

[evo14348-bib-0035] Goodnight, K. F. , and D. C. Queller . 1999. Computer software for performing likelihood tests of pedigree relationship using genetic markers. Mol. Ecol 8:1231–1234. 10447863 10.1046/j.1365-294x.1999.00664.x

[evo14348-bib-0036] Green, J. P. , and B. J. Hatchwell . 2018. Inclusive fitness consequences of dispersal decisions in a cooperatively breeding bird, the long‐tailed tit (*Aegithalos caudatus*). Proc. Natl. Acad. Sci. U.S.A 115:12011–12016. 30397131 10.1073/pnas.1815873115 PMC6255206

[evo14348-bib-0037] Griffin, A. S. , and S. A. West . 2003. Kin discrimination and the benefit of helping in cooperatively breeding vertebrates. Science 302:634–636. 14576431 10.1126/science.1089402

[evo14348-bib-0038] Griffin, A. S. , and S. A. West . 2002. Kin selection: fact and fiction. Trends Ecol. Evol 17:15–21.

[evo14348-bib-0039] Groenewoud, F. , J. G. Frommen , D. Josi , H. Tanaka , A. Jungwirth , and M. Taborsky . 2016. Predation risk drives social complexity in cooperative breeders. Proc. Natl. Acad. Sci. U.S.A 113:4104–4109. 27035973 10.1073/pnas.1524178113 PMC4839406

[evo14348-bib-0040] Hamilton, I. M. , and I. Y. Ligocki . 2012. The extended personality: indirect effects of behavioural syndromes on the behaviour of others in a group‐living cichlid. Anim. Behav 84:659–664.

[evo14348-bib-0041] Hamilton, W. D. 1964. The genetical evolution of social behavior, parts I and II. J. Theor. Biol 7:1–52. 5875341 10.1016/0022-5193(64)90038-4

[evo14348-bib-0042] Heg, D. , and Z. Bachar . 2006. Cooperative breeding in the Lake Tanganyika cichlid *Julidochromis ornatus* . Environ. Biol. Fishes 76:265–281.

[evo14348-bib-0043] Heg, D. , Z. Bachar , L. Brouwer , and M. Taborsky . 2004. Predation risk is an ecological constraint for helper dispersal in a cooperatively breeding cichlid. Proc. R. Soc. B 271:2367–2374. 10.1098/rspb.2004.2855 PMC1691868 15556889

[evo14348-bib-0044] Heg, D. , Z. Bachar , and M. Taborsky . 2005a. Cooperative breeding and group structure in the Lake Tanganyika cichlid *Neolamprologus savoryi* . Ethology 111:1017–1043.

[evo14348-bib-0045] Heg, D. , R. Bergmüller , D. Bonfils , O. Otti , Z. Bachar , R. Burri , G. Heckel , and M. Taborsky . 2006. Cichlids do not adjust reproductive skew to the availability of independent breeding options. Behav. Ecol 17:419–429.

[evo14348-bib-0046] Heg, D. , L. Brouwer , Z. Bachar , and M. Taborsky . 2005b. Large group size yields group stability in the cooperatively breeding cichlid *Neolamprologus pulcher* . Behaviour 142:1615–1641.

[evo14348-bib-0047] Heg, D. , and I. M. Hamilton . 2008. Tug‐of‐war over reproduction in a cooperatively breeding cichlid. Behav. Ecol. Sociobiol 62:1249–1257.

[evo14348-bib-0048] Heg, D. , Z. Heg‐Bachar , L. Brouwer , and M. Taborsky . 2008. Experimentally induced helper dispersal in colonially breeding cooperative cichlids. Environ. Biol. Fishes 83:191–206.

[evo14348-bib-0049] Hellmann, J. K. , I. Y. Ligocki , C. M. O'Connor , A. R. Reddon , K. A. Garvy , S. E. Marsh‐Rollo , H. Lisle Gibbs , S. Balshine , and I. M. Hamilton . 2015. Reproductive sharing in relation to group and colony‐level attributes in a cooperative breeding fish. Proc. R. Soc. B 282:20150954. 10.1098/rspb.2015.0954 PMC4528555 26136450

[evo14348-bib-0050] Hellmann, J. K. , M. G. Sovic , H. L. Gibbs , A. R. Reddon , C. M. O'Connor , I. Y. Ligocki , S. Marsh‐Rollo , S. Balshine , and I. M. Hamilton . 2016. Within‐group relatedness is correlated with colony‐level social structure and reproductive sharing in a social fish. Mol. Ecol 25:4001–4013. 27297293 10.1111/mec.13728

[evo14348-bib-0051] Hultgren, K. M. , and J. E. Duffy . 2012. Phylogenetic community ecology and the role of social dominance in sponge‐dwelling shrimp. Ecol. Lett 15:704–713. 22548770 10.1111/j.1461-0248.2012.01788.x

[evo14348-bib-0052] Jamieson, I. G. 1997. Testing reproductive skew models in a communally breeding bird, the pukeko, *Porhyrio porphyrio* . Proc. R. Soc. B 264:335–340.

[evo14348-bib-0053] Johnstone, R. A. 2000. Models of reproductive skew: a review and synthesis. Ethology 106:5–26.

[evo14348-bib-0054] Jordan, L. A. , S. M. Maguire , H. A. Hofmann , and M. Kohda . 2016. The social and ecological costs of an ‘over‐extended’ phenotype. Proc. R. Soc. B 283:20152359. 10.1098/rspb.2015.2359 PMC4721094 26740619

[evo14348-bib-0055] Josi, D. , M. Taborsky , and J. G. Frommen . 2019. First field evidence for alloparental egg care in cooperatively breeding fish. Ethology 125:164–169.

[evo14348-bib-0056] Josi, D. , A. Freudiger , M. Taborsky , and J. G. Frommen . 2020a. Experimental predator intrusions in a cooperative breeder reveal threat‐dependent task partitioning. Behav. Ecol 31:1369–1378.

[evo14348-bib-0057] Josi, D. , M. Taborsky , and J. G. Frommen . 2020b. Investment of group members is contingent on helper number and the presence of young in a cooperative breeder. Anim. Behav 160:35–42.

[evo14348-bib-0257] Josi, D. , J. M. Flury , M. Reyes‐Contreras , H. Tanaka , M. Taborsky , and J. G. Frommen . 2021. Sex‐Specific Routes to Independent Breeding in a Polygynous Cooperative Breeder. Front. Ecol. Evol 9:750483.

[evo14348-bib-0059] Jungwirth, A. , and M. Taborsky . 2015. First‐ and second‐order sociality determine survival and reproduction in cooperative cichlids. Proc. R. Soc. B 282:20151971. 10.1098/rspb.2015.1971 PMC4685815 26582022

[evo14348-bib-0060] Jungwirth, A. , M. Zöttl , D. Bonfils , D. Josi , J. G. Frommen , and M. Taborsky . (in revision). Sex‐specific life history trajectories and the fitness effects of dispersal in a cooperatively breeding fish. 10.1126/sciadv.add2146 PMC9984175 36867697

[evo14348-bib-0061] Kasper, C. , M. Kölliker , E. Postma , and B. Taborsky . 2017. Consistent cooperation in a cichlid fish is caused by maternal and developmental effects rather than heritable genetic variation. Proc. R. Soc. B 284:20170369. 10.1098/rspb.2017.0369 PMC5524489 28701555

[evo14348-bib-0062] Keller, L. , and H. K. Reeve . 1994. Partitioning of reproduction in animal societies. Trends Ecol. Evol 9:98–102. 21236786 10.1016/0169-5347(94)90204-6

[evo14348-bib-0063] Kingma, S. A. 2017. Direct benefits explain interspecific variation in helping behaviour among cooperatively breeding birds. Nat. Commun 8:1094. 29061969 10.1038/s41467-017-01299-5 PMC5653647

[evo14348-bib-0064] Kingma, S. A. , M. L. Hall , and A. Peters . 2011. Multiple benefits drive helping behavior in a cooperatively breeding bird: an integrated analysis. Am. Nat 177:486–495. 21460570 10.1086/658989

[evo14348-bib-0065] Kingma, S. A. , P. Santema , M. Taborsky , and J. Komdeur . 2014. Group augmentation and the evolution of cooperation. Trends Ecol. Evol 29:476–484. 24996259 10.1016/j.tree.2014.05.013

[evo14348-bib-0066] Koenig, W. D. , and J. L. Dickinson . 2016. Cooperative breeding in vertebrates. Cambridge University Press, Cambridge.

[evo14348-bib-0067] Koenig, W. D. , S. F. Shen , A. H. Krakauer , and J. Haydock . 2009. Reproductive skew in avian societies. Pp. 227–264 *in* R. Hager and C. B. Jones , eds. Reproductive skew in vertebrates: proximate and ultimate causes. Cambridge University Press, Cambridge.

[evo14348-bib-0068] Kohda, M. , D. Heg , Y. Makino , T. Takeyama , J. Shibata , K. Watanabe , H. Munehara , M. Hori , and S. Awata . 2009. Living on the wedge: female control of paternity in a cooperatively polyandrous cichlid. Proc. R. Soc. B 276:4207–4214. 10.1098/rspb.2009.1175 PMC2821345 19726479

[evo14348-bib-0069] Kohler, U. 1998. Zur Struktur und Evolution des Sozialsystems von *Neolamprologus multifasciatus* (Cichlidae, Pisces), dem kleinsten Schneckenbuntbarsch des Tanganjikasees. Shaker Verlag, Aachen.

[evo14348-bib-0070] Kokko, H. , R. A. Johnstone , and J. Wright . 2002. The evolution of parental and alloparental effort in cooperatively breeding groups: when should helpers pay to stay? Behav. Ecol 13:291–300.

[evo14348-bib-0071] Komdeur, J. , T. Burke , H. L. Dugdale , and D. S. Richardson . 2016. Seychelles warblers: complexities of the helping paradox. Pp. 197–216 *in* W. D. Koenig and J. L. Dickinson , eds. Cooperative breeding in vertebrates: studies of ecology, evolution, and behavior. Cambridge University Press, Cambridge.

[evo14348-bib-0072] Komdeur, J. , D. S. Richardson , M. Hammers , C. Eikenaar , L. Brouwer , and S. A. Kingma . 2017. The evolution of cooperative breeding in vertebrates. Pp. 1–11 *in* eLS. John Wiley & Sons, Ltd, Chichester.

[evo14348-bib-0073] Konovalov, D. A. 2006. Accuracy of four heuristics for the full sibship reconstruction problem in the presence of genotype errors. Pp. 7–16 *in* Proceedings of the 4th Asia‐Pacific Bioinformatics Conference. Imperial College Press, London.

[evo14348-bib-0074] Konovalov, D. A. , and D. Heg . 2008. Estimation of population allele frequencies from small samples containing multiple generations. Pp. 321–332 *in* Proceedings of the 6th Asia‐Pacific Bioinformatics Conference. Imperial College Press, London.

[evo14348-bib-0075] Konovalov, D. A. , C. Manning , and M. T. Henshaw . 2004. KINGROUP: A program for pedigree relationship reconstruction and kin group assignments using genetic markers. Mol. Ecol. Notes 4:779–782.

[evo14348-bib-0076] Kreiberg, H. 2000. Stress and Anesthesia. Pp. 503–511 *in* G. Ostrander , ed. The Laboratory Fish. Academic Press, New York.

[evo14348-bib-0077] Le Vin, A. L. , B. K. Mable , M. Taborsky , D. Heg , and K. E. Arnold . 2011. Individual variation in helping in a cooperative breeder: relatedness versus behavioural type. Anim. Behav 82:467–477.

[evo14348-bib-0078] Leadbeater, E. , J. M. Carruthers , J. P. Green , N. S. Rosser , and J. Field . 2011. Nest inheritance is the missing source of direct fitness in a primitively eusocial insect. Science 333:874–876. 21836014 10.1126/science.1205140

[evo14348-bib-0079] Leedale, A. E. , S. P. Sharp , M. Simeoni , E. J. H. Robinson , and B. J. Hatchwell . 2018. Fine‐scale genetic structure and helping decisions in a cooperatively breeding bird. Mol. Ecol 27:1714–1726. 29543401 10.1111/mec.14553

[evo14348-bib-0080] Lucas, E. R. , R. P. Martins , and J. Field . 2011. Reproductive skew is highly variable and correlated with genetic relatedness in a social apoid wasp. Behav. Ecol 22:337–344.

[evo14348-bib-0081] Lukas, D. , and T. Clutton‐Brock . 2018. Social complexity and kinship in animal societies. Ecol. Lett 21:1129–1134. 29797749 10.1111/ele.13079

[evo14348-bib-0082] Maan, M. E. , and M. Taborsky . 2008. Sexual conflict over breeding substrate causes female expulsion and offspring loss in a cichlid fish. Behav. Ecol 19:302–308.

[evo14348-bib-0083] Naef, J. , and M. Taborsky . 2020a. Commodity‐specific punishment for experimentally induced defection in cooperatively breeding fish. R. Soc. Open Sci 7:191808. 32257335 10.1098/rsos.191808 PMC7062066

[evo14348-bib-0084] Naef, J. , and M. Taborsky . 2020b. Punishment controls helper defence against egg predators but not fish predators in cooperatively breeding cichlids. Anim. Behav 168:137–147.

[evo14348-bib-0085] Nelson‐Flower, M. J. , P. A. R. Hockey , C. O'Ryan , and A. R. Ridley . 2012. Inbreeding avoidance mechanisms: dispersal dynamics in cooperatively breeding southern pied babblers. J. Anim. Ecol 81:876–883. 22471769 10.1111/j.1365-2656.2012.01983.x

[evo14348-bib-0086] Nichols, H. J. 2017. The causes and consequences of inbreeding avoidance and tolerance in cooperatively breeding vertebrates. J. Zool 303:1–14.

[evo14348-bib-0087] Painter, J. N. , R. H. Crozier , A. Poiani , R. J. Robertson , and M. F. Clarke . 2000. Complex social organization reflects genetic structure and relatedness in the cooperatively breeding bell miner, *Manorina melanophrys* . Mol. Ecol 9:1339–1347. 10972773 10.1046/j.1365-294x.2000.01012.x

[evo14348-bib-0403] Parker, A. , and I. Kornfield . 1996. Polygynandry in *Pseudotropheus zebra*, a cichlid fish from Lake Malawi. Environ. Biol. Fishes 47:345–352.

[evo14348-bib-0088] Quiñones, A. E. , G. S. van Doorn , I. Pen , F. J. Weissing , and M. Taborsky . 2016. Negotiation and appeasement can be more effective drivers of sociality than kin selection. Philos. Trans. R. Soc. B 371:20150089. 10.1098/rstb.2015.0089 PMC4760191 26729929

[evo14348-bib-0089] R core team. 2017. R: A language and environment for statistical computing. R Found. Stat. Comput. Vienna, Austria https://www.r‐project.org/ Vienna, Austria.

[evo14348-bib-0090] Raihani, N. J. , and T. H. Clutton‐Brock . 2010. Higher reproductive skew among birds than mammals in cooperatively breeding species. Biol. Lett 6:630–632. 20236970 10.1098/rsbl.2010.0159 PMC2936150

[evo14348-bib-0091] Reeve, H. K. , and L. Keller . 1997. Reproductive bribing and policing as evolutionary mechanisms for the suppression of within‐group selfishness. Am. Nat 150:542–550. 10.1086/286049 18811311

[evo14348-bib-0092] Reeve, H. K. , and L. Keller . 2001. Tests of reproductive‐skew models in social insects. Annu. Rev. Entomol 46:347–385. 11112173 10.1146/annurev.ento.46.1.347

[evo14348-bib-0093] Reeve, H. K. , P. T. Starks , J. M. Peters , and P. Nonacs . 2000. Genetic support for the evolutionary theory of reproductive transactions in social wasps. Proc. R. Soc. B 267:75–79. 10.1098/rspb.2000.0969 PMC1690491 10670956

[evo14348-bib-0094] Richardson, D. S. , T. Burke , and J. Komdeur . 2002. Direct benefits and the evolution of female‐biased cooperative breeding in Seychelles warblers. Evolution 56:2313–2321. 12487360 10.1111/j.0014-3820.2002.tb00154.x

[evo14348-bib-0095] Riehl, C. 2013. Evolutionary routes to non‐kin cooperative breeding in birds. Proc. R. Soc. B 280:20132245. 10.1098/rspb.2013.2245 PMC3813341 24132311

[evo14348-bib-0096] Riehl, C. 2017. Kinship and incest avoidance drive patterns of reproductive skew in cooperatively breeding birds. Am. Nat 190:774–785. 29166167 10.1086/694411

[evo14348-bib-0097] Ronco, F. , M. Matschiner , A. Böhne , A. Boila , H. H. Büscher , A. El Taher , A. Indermaur , M. Malinsky , V. Ricci , A. Kahmen , et al. 2021. Drivers and dynamics of a massive adaptive radiation in cichlid fishes. Nature 589:76–81. 33208944 10.1038/s41586-020-2930-4

[evo14348-bib-0098] Russell, A. F. , and B. J. Hatchwell . 2001. Experimental evidence for kin‐biased helping in a cooperatively breeding vertebrate. Proc. R. Soc. B 268:2169–2174. 10.1098/rspb.2001.1790 PMC1088862 11600082

[evo14348-bib-0099] Santema, P. , and T. Clutton‐Brock . 2013. Meerkat helpers increase sentinel behaviour and bipedal vigilance in the presence of pups. Anim. Behav 85:655–661.

[evo14348-bib-0399] Schliewen, U. , K. Rassmann , M. Markmann , J. Markert , T. Kocher , and D. Tautz . 2021. Genetic and ecological divergence of a monophyletic cichlid species pair under fully sympatric conditions in Lake Ejagham, Cameroon. Mol. Ecol 10:1471–1488. 10.1046/j.1365-294x.2001.01276.x 11412369

[evo14348-bib-0100] Shen, S. F. , H. W. Yuan , and M. Liu . 2016. Taiwan yuhinas: Unrelated joint‐nesters cooperate in unfavorable environments. Pp. 237–256 *in* W. D. Koenig and J. L. Dickinson , eds. Cooperative breeding in vertebrates: studies of ecology, evolution, and behavior. Cambridge University Press, Cambridge.

[evo14348-bib-0101] Skubic, E. , M. Taborsky , J. M. McNamara , and A. I. Houston . 2004. When to parasitize? A dynamic optimization model of reproductive strategies in a cooperative breeder. J. Theor. Biol 227:487–501. 15038984 10.1016/j.jtbi.2003.11.021

[evo14348-bib-0102] Solomon, N. G. , and J. A. French . 1997. Cooperative breeding in mammals. Cambridge University Press, Cambridge.

[evo14348-bib-0103] Stiver, K. A. , P. Dierkes , M. Taborsky , H. L. Gibbs , and S. Balshine . 2005. Relatedness and helping in fish: examining the theoretical predictions. Proc. R. Soc. B 272:1593–1599. 10.1098/rspb.2005.3123 PMC1559835 16048775

[evo14348-bib-0104] Stiver, K. A. , J. Fitzpatrick , J. Desjardins , and S. Balshine . 2006. Sex differences in rates of territory joining and inheritance in a cooperatively breeding cichlid fish. Anim. Behav 71:449–456.

[evo14348-bib-0105] Taborsky, M. 1984. Broodcare helpers in the cichlid fish *Lamprologus brichardi*: their costs and benefits. Anim. Behav 32:1236–1252.

[evo14348-bib-0106] Taborsky, M. 1994. Sneakers, satellites, and helpers: parasitic and cooperative behavior in fish reproduction. Adv. Study Behav 23:1–100.

[evo14348-bib-0107] Taborsky, M. 2009. Reproductive skew in cooperative fish groups: virtue and limitations of alternative modeling approaches. Pp. 265–304 *in* R. Hager and C. B. Jones , eds. Reproductive skew in vertebrates: proximate and ultimate causes. Cambridge University Press, Cambridge.

[evo14348-bib-0108] Taborsky, M. 2016. Cichlid fishes: a model for the integrative study of social behavior. Pp. 272–293 *in* W. D. Koenig and J. L. Dickinson , eds. Cooperative breeding in vertebrates: studies of ecology, evolution, and behavior. Cambridge University Press, Cambridge.

[evo14348-bib-0109] Taborsky, M. , and D. Limberger . 1981. Helpers in fish. Behav. Ecol. Sociobiol 8:143–145.

[evo14348-bib-0110] Taborsky, M. , J. G. Frommen , and C. Riehl . 2016. Correlated pay‐offs are key to cooperation. Philos. Trans. R. Soc. B 371:20150084. 10.1098/rstb.2015.0084 PMC4760186 26729924

[evo14348-bib-0111] Tanaka, H. , J. G. Frommen , L. Engqvist , and M. Kohda . 2018a. Task‐dependent workload adjustment of female breeders in a cooperatively breeding fish. Behav. Ecol 29:221–229.

[evo14348-bib-0112] Tanaka, H. , J. G. Frommen , S. Koblmüller , K. M. Sefc , M. McGee , M. Kohda , S. Awata , M. Hori , and M. Taborsky . 2018b. Evolutionary transitions to cooperative societies in fishes revisited. Ethology 124:777–789.

[evo14348-bib-0113] Tanaka, H. , J. G. Frommen , T. Takahashi , and M. Kohda . 2016. Predation risk promotes delayed dispersal in the cooperatively breeding cichlid *Neolamprologus obscurus* . Anim. Behav 117:51–58.

[evo14348-bib-0114] Tanaka, H. , D. Heg , H. Takeshima , T. Takeyama , S. Awata , M. Nishida , and M. Kohda . 2015. Group composition, relatedness, and dispersal in the cooperatively breeding cichlid *Neolamprologus obscurus* . Behav. Ecol. Sociobiol 69:169–181.

[evo14348-bib-0115] Teunissen, N. , S. A. Kingma , and A. Peters . 2020. Predator defense is shaped by risk, brood value and social group benefits in a cooperative breeder. Behav. Ecol 31:761–771.

[evo14348-bib-0116] Trapote, E. , D. Canestrari , and V. Baglione . 2021. Female helpers signal their contribution to chick provisioning in a cooperatively breeding bird. Anim. Behav 172:113–120.

[evo14348-bib-0401] Van Oppen, M. H. , C. Rico , J. C. Deutsch , G. F. Turner , and G. M. Hewitt . 1997. Isolation and characterization of microsatellite loci in the cichlid fish *Pseudotropheus zebra* . Mol. Ecol 6:387–388. 9131814 10.1046/j.1365-294x.1997.00188.x

[evo14348-bib-0117] Vehrencamp, S. L. 1983. Optimal degree of skew in cooperative societies. Amer. Zool 23:327–335.

[evo14348-bib-0118] Vitikainen, E. I. K. , H. H. Marshall , F. J. Thompson , J. L. Sanderson , M. B. V. Bell , J. S. Gilchrist , S. J. Hodge , H. J. Nichols , and M. A. Cant . 2017. Biased escorts: offspring sex, not relatedness explains alloparental care patterns in a cooperative breeder. Proc. R. Soc. B 284:20162384. 10.1098/rspb.2016.2384 PMC5443930 28469015

[evo14348-bib-0119] Wikberg, E. C. , K. M. Jack , L. M. Fedigan , F. A. Campos , A. S. Yashima , M. L. Bergstrom , T. Hiwatashi , and S. Kawamura . 2017. Inbreeding avoidance and female mate choice shape reproductive skew in capuchin monkeys (*Cebus capucinus imitator*). Mol. Ecol 26:653–667. 27797426 10.1111/mec.13898

[evo14348-bib-0402] Zardoya, R. , D. M. Vollmer , C. Craddock , J. T. Streelman , S. Karl , and A. Meyer . 1996. Evolutionary conservation of microsatellite flanking regions and their use in resolving the phylogeny of cichlid fishes (Pisces: Perciformes). Proc. R. Soc. B 263:1589–1598. 10.1098/rspb.1996.0233 8952095

[evo14348-bib-0120] Zöttl, M. , D. Heg , N. Chervet , and M. Taborsky . 2013. Kinship reduces alloparental care in cooperative cichlids where helpers pay‐to‐stay. Nat. Commun 4:1341. 23299891 10.1038/ncomms2344

